# How adolescent cannabis use reshapes the developing brain — a systematic review

**DOI:** 10.3389/fpsyt.2026.1822300

**Published:** 2026-04-27

**Authors:** Valerio Ricci, Alessandro Sarni, Domenico De Berardis, Giovanni Martinotti, Giuseppe Maina

**Affiliations:** 1 San Luigi Gonzaga Hospital, University of Turin, Orbassano, Italy; 2 Department of Neurosciences “Rita Levi Montalcini”, University of Turin, Turin, Italy; 3 Department of Mental Health, Psychiatric Service for Diagnosis and Treatment, Hospital “G. Mazzini”, ASL 4, Teramo, Italy; 4 Department of Neurosciences, Imaging and Clinical Sciences, Università degli Studi G. D’Annunzio Chieti-Pescara, Chieti, Italy

**Keywords:** addiction vulnerability, adolescent cannabis use, brain imaging, cognitive effects, neurodevelopment

## Abstract

**Background and hypothesis:**

Cannabis use initiation during adolescence has increased globally, raising concerns about neurodevelopmental consequences during this critical period when the brain undergoes extensive remodeling in cannabinoid receptor-rich regions.

**Study design:**

This systematic review examines neurodevelopmental consequences of adolescent cannabis use, focusing on structural brain changes, cognitive impacts, addiction vulnerability, and long-term outcomes. We searched PubMed, EMBASE, PsycINFO, and Web of Science (2000-2025) for studies examining cannabis effects in adolescent populations. Following PRISMA guidelines, two reviewers screened 3,421 records and assessed 156 full-text articles, including studies with neuroimaging, cognitive assessments, or longitudinal follow-up.

**Study results:**

Thirty-six studies involving 8,432 participants met criteria: 23 longitudinal cohorts (62.2%), 8 cross-sectional (22.2%), 4 RCTs (11.1%), and 1 case-control study (2.8%). Neuroimaging revealed dose-dependent alterations including reduced prefrontal cortical and hippocampal/amygdala volumes, accelerated cortical thinning in longitudinal studies, and impaired white matter connectivity correlating with initiation age. Cognitive findings were mixed — some showed persistent deficits after prolonged abstinence in adolescent-onset users, others found no effects after controlling for confounders. Epidemiological studies consistently showed elevated addiction risk (ORs 3.9–7.2) in adolescents versus adults. Long-term associations included educational difficulties, mental health problems, and functional impairment, though causal relationships remained unclear.

**Conclusions:**

Adolescent cannabis use associates with structural brain changes, elevated addiction risk, and variable cognitive effects, suggesting greater vulnerability versus adult-onset use. However, methodological limitations including confounders, heterogeneous definitions, and observational designs limit causal inference. Findings support age-specific prevention and specialized interventions while highlighting needs for rigorous longitudinal research establishing causality.

**Systematic review registration:**

https://www.crd.york.ac.uk/prospero/, identifierCRD420251165329.

## 1 Introduction

The human brain undergoes extensive remodeling during adolescence, with prefrontal cortex maturation continuing into the mid-twenties (1, 2). This extended developmental period involves synaptic pruning, white matter myelination, and circuit refinement processes that create both opportunities for learning and windows of vulnerability to environmental influences (3, 4).

The endocannabinoid system plays critical roles in adolescent neurodevelopment, regulating neuronal migration, axon guidance, and synaptic pruning (5). Cannabinoid CB1 receptors show peak expression in adolescent prefrontal and limbic regions during active developmental periods, creating potential windows of heightened sensitivity to cannabis exposure (6).

Cannabis use among adolescents has increased significantly over the past two decades, with approximately 35% of U.S. high school seniors reporting past-year use in recent national surveys, and the average age of initiation decreasing to 14.6 years compared to estimates of 17–18 years in earlier cohort studies (7–9). Concurrent increases in cannabis potency, with contemporary products containing 15-25% THC compared to 3-4% in previous decades, may amplify risks for developing psychosis and other adverse neuropsychiatric outcomes (7). Changing legal frameworks and decreased risk perception among adolescents have contributed to this trend, creating an urgent need to understand the neurodevelopmental consequences of adolescent cannabis exposure (8–10). Emerging evidence suggests that both timing and frequency of cannabis exposure during adolescence are critical determinants of neurodevelopmental risk (11), with frequency of use potentially mediating the relationship between early initiation and adverse outcomes (12). The “critical period hypothesis” proposes that cannabis use during adolescence produces qualitatively different and more severe effects compared to adult-onset use (13–15). This is supported by adolescent-specific brain vulnerability, distinct CB1 receptor developmental patterns, and epidemiological evidence showing worse outcomes with earlier versus later onset (10, 16, 17). Studies have documented structural brain alterations, cognitive deficits, and increased addiction risk specifically associated with adolescent-onset cannabis use (18), with some effects persisting years after cessation (19, 20). However, findings are not entirely consistent, with some well-controlled studies questioning causal relationships and highlighting the role of confounding factors (21, 22).

Despite growing research interest, significant gaps remain in our understanding of adolescent cannabis effects. Many studies are cross-sectional or have limited follow-up periods, and heterogeneity in methodologies complicates synthesis. The observational nature of most research limits causal inference, as early cannabis users often differ from non-users across multiple domains, including pre-existing psychiatric vulnerabilities, adverse childhood experiences, family history of substance use disorders, socioeconomic disadvantage, and co-occurring use of other substances.

These knowledge gaps have important implications for clinical practice and policy. Clinicians need evidence-based guidance for adolescent risk assessment and treatment, while policy debates require clear understanding of developmental vulnerability to inform age-based restrictions and prevention approaches.

Given increasing adolescent cannabis use, changing legal frameworks, and emerging evidence for developmental vulnerability, a comprehensive synthesis of current knowledge is urgently needed. While previous reviews have examined aspects of adolescent cannabis effects, no recent systematic review has comprehensively evaluated evidence for developmental timing effects across multiple domains.

This systematic review aims to synthesize current evidence on the neurodevelopmental consequences of adolescent cannabis use, with particular focus on how effects differ by age of initiation. Our specific objectives are to: Examine neuroimaging evidence of structural and functional brain changes associated with adolescent cannabis use; Evaluate cognitive consequences and recovery patterns following adolescent versus adult-onset cannabis use; Assess addiction vulnerability and progression patterns in adolescent-onset users; Analyze long-term functional outcomes including educational, occupational, and mental health trajectories; Identify biological mechanisms underlying developmental vulnerability and individual differences; Evaluate methodological quality and identify limitations in current evidence; Provide evidence-based recommendations for clinical practice, prevention, and future research priorities.

By comprehensively reviewing evidence across these domains, this systematic review aims to inform evidence-based approaches to adolescent cannabis prevention and intervention while identifying critical areas for future investigation.

## 2 Methods

### 2.1 Search strategy

**Figure 1:**
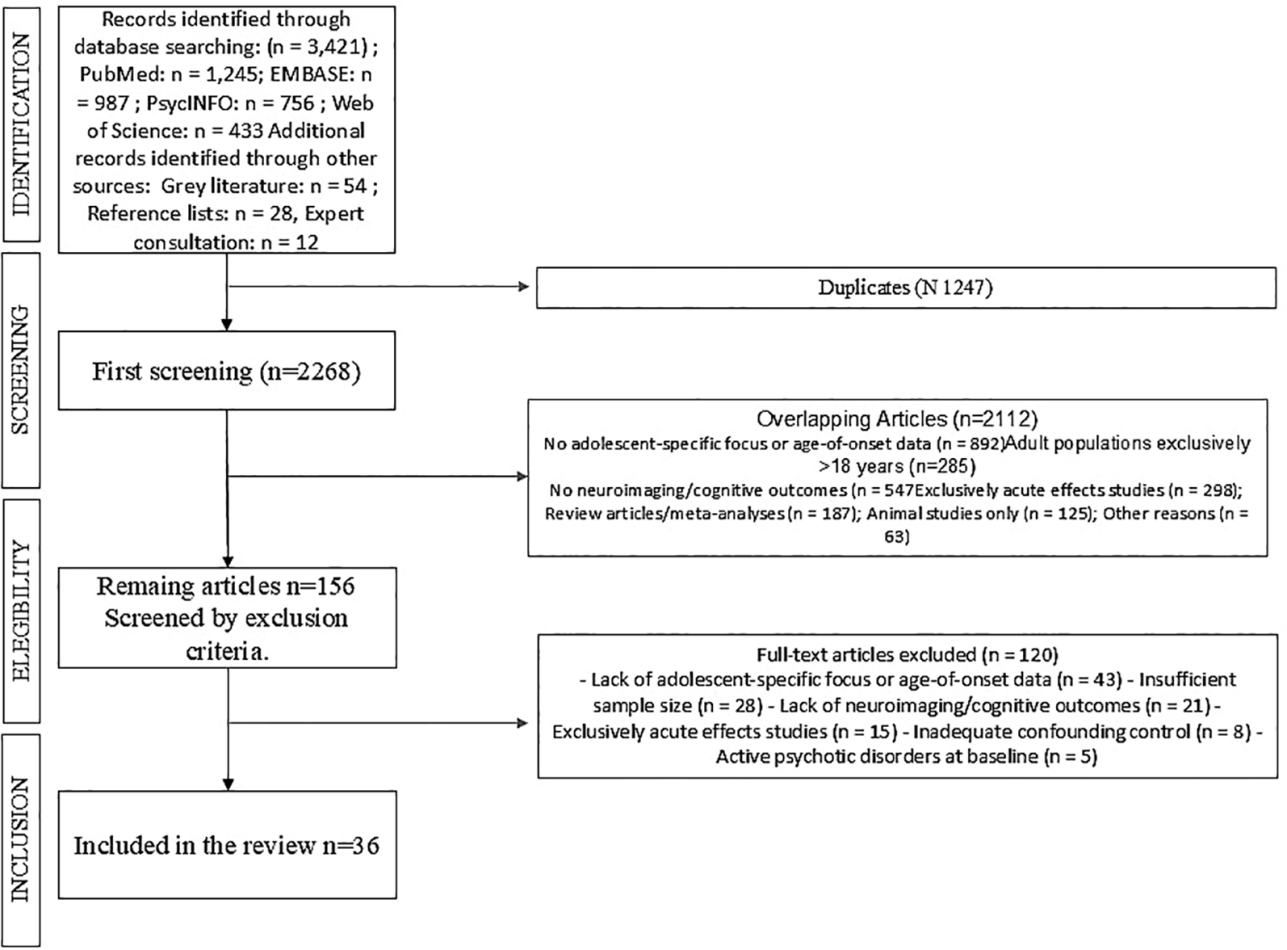
Flow-chart of study search and selection process.

This systematic review was conducted following the Preferred Reporting Items for Systematic Reviews and Meta-Analyses (PRISMA) guidelines (23)(**Appendix A**; **Figure 1**). The protocol for this systematic review was registered with PROSPERO (International Prospective Register of Systematic Reviews) (CRD420251165329) A comprehensive literature search was conducted using PubMed, EMBASE, PsycINFO, and Cochrane databases from January 2000 to September 2025.

The search strategy employed a combination of Medical Subject Headings (MeSH) terms and free-text keywords related to: (1) cannabis exposure (“cannabis,” “marijuana,” “THC,” “cannabinoid,” “hashish”); (2) adolescent populations (“adolescent,” “teenager,” “youth,” “juvenile,” “young adult,” “developmental”); (3) neurodevelopmental outcomes (“neurodevelopment,” “brain development,” “neuroimaging,” “MRI,” “cognitive function,” “executive function,” “memory,” “intelligence”); and (4) timing factors (“early onset,” “age of initiation,” “adolescent-onset,” “developmental timing”).

Search terms were combined using Boolean operators (AND/OR) and adapted for each database’s specific syntax. Additionally, we conducted manual searches of reference lists from included studies and relevant review articles to identify additional eligible studies. Grey literature was searched through conference proceedings from major neuroscience and addiction conferences (Society for Neuroscience, Research Society on Alcoholism, International Cannabis Research Society) and doctoral dissertation databases (ProQuest Dissertations & Theses Global) (**Appendix B**).

### 2.2 Study selection and eligibility criteria

#### 2.2.1 Inclusion criteria

Population: Studies examining adolescent cannabis users (age of first use ≤18 years) or comparing adolescent-onset versus adult-onset cannabis users.

Exposure: Clear definition of cannabis use patterns, frequency, duration, or age of initiation.

Outcomes: Included at least one of the following measures:

-Structural or functional neuroimaging (MRI, fMRI, DTI, PET)
-Cognitive assessment using standardized neuropsychological tests
-Longitudinal behavioral or functional outcomes (academic, occupational, mental health)
-Biological measures related to neurodevelopment (receptor expression, neurotransmitter function)

Study design: Cross-sectional comparisons, longitudinal cohort studies, or intervention studies.

Sample size: Studies with adequate statistical power to detect clinically meaningful differences. For comparative studies, minimum 15 participants per group; for longitudinal studies, minimum 30 participants at baseline. Studies with smaller samples were considered if they presented particularly innovative methodologies or unique assessments not available in larger studies. All included studies met the minimum sample size criteria. For studies with total samples near the threshold (e.g., n=33), group distributions were verified to ensure adequate power in each comparison group (≥15 per group for case-control or cross-sectional designs).

Confounding control: Studies that controlled for or assessed key confounding variables including alcohol use, other substance use, and socioeconomic factors, or that provided sufficient data to evaluate the influence of these factors.

Publication: Peer-reviewed articles published in English-language journals, plus high-quality grey literature (doctoral dissertations, government reports, conference abstracts) meeting rigorous methodological criteria.

Methodological quality: Studies with clearly defined methodology and appropriate statistical analyses. Pilot studies or those with methodological limitations were included if they provided unique contributions to understanding adolescent cannabis use effects, with appropriate discussion of limitations in results interpretation.

Animal studies were included exclusively when they provided direct mechanistic evidence pertaining to endocannabinoid system development during adolescence that could not be derived from available human studies. Given the focus of this review on human neurodevelopmental outcomes, preclinical studies were considered only as supplementary biological context and were therefore limited to studies most directly informing the critical period hypothesis examined her.

#### 2.2.2 Exclusion criteria

Studies were excluded if they:

-Focused exclusively on acute intoxication effects or withdrawal symptoms without examining chronic use effects
-Included participants with active psychotic disorders or severe psychiatric conditions at baseline that could confound neurodevelopmental measures
-Lacked clear operational definitions of cannabis use patterns or age of initiation
- Had follow-up periods less than 6 months for observational longitudinal cohort studies (this criterion did not apply to randomized controlled trials, where shorter intervention periods were acceptable given the experimental design and controlled conditions)
-Were case reports, editorials, reviews, or studies without original data
-Used exclusively preclinical animal models without human validation (except for mechanistic studies providing biological context)
-Failed to adequately control for or assess major confounding variables
-Had significant methodological limitations that compromised data quality or interpretation.

### 2.3 Data extraction and management

Two independent reviewers (initials) conducted the initial screening of titles and abstracts using predefined eligibility criteria. Full-text articles of potentially eligible studies were then independently reviewed by both reviewers. Disagreements were resolved through discussion, and when necessary, a third reviewer was consulted.

Data extraction was performed systematically using a standardized data extraction form that captured:

-Study characteristics: First author, publication year, study design, country, sample size, demographic characteristics
-Cannabis use variables: Age of initiation, frequency of use, duration of use, potency measures (when available), abstinence periods
-Comparison groups: Control group characteristics, matching criteria, group definitions
-Outcomes: Neuroimaging findings (structural volumes, connectivity measures, activation patterns), cognitive test results, functional outcomes, biological measures
-Methodology: Neuroimaging protocols, cognitive assessment batteries, statistical approaches, confounding variable control
-Results: Effect sizes, confidence intervals, p-values, direction of associations (**Appendix B**).

### 2.4 Quality assessment and risk of bias

Study quality was assessed independently by two reviewers using the Newcastle-Ottawa Scale (NOS) (21)adapted for cross-sectional and cohort studies (**Appendix C**). The assessment evaluated three domains:

-Selection of study groups: Representativeness of exposed cohort, selection of non-exposed cohort, ascertainment of exposure
-Comparability of groups: Comparability on the basis of design or analysis controlled for confounders
-Assessment of outcomes: Assessment of outcome, adequacy of follow-up (for cohort studies)

Studies were rated as good quality (≥7 stars), fair quality (4–6 stars), or poor quality (≤3 stars). For intervention studies, the Cochrane Risk of Bias tool was used to assess randomization, allocation concealment, blinding, incomplete outcome data, selective reporting, and other potential sources of bias.

Additional sources of bias specifically assessed included:

-Selection bias: Differences between cannabis users and controls in baseline characteristics
-Information bias: Recall bias in self-reported cannabis use, measurement error in neuroimaging or cognitive testing
-Confounding bias: Inadequate control for alcohol, tobacco, other substances, socioeconomic status, genetic factors
-Publication bias: Assessment through funnel plot examination and Egger’s test when sufficient studies were available

### 2.5 Data synthesis and analysis

Given the heterogeneity in study designs, cannabis use definitions, and outcome measures, a narrative synthesis approach was employed rather than formal meta-analysis. Studies were grouped thematically by: Neuroimaging findings (structural and functional); Cognitive and academic outcomes; Long-term functional outcomes; Biological mechanisms and individual differences.

For studies reporting similar outcomes with comparable methodologies, effect sizes were calculated and compared qualitatively. When possible, standardized mean differences were computed to facilitate comparison across studies.

Subgroup analyses were conducted based on:

-Age of cannabis initiation (early adolescence ≤16 years vs. late adolescence 17–18 years vs. adult onset >18 years)
-Frequency and intensity of use (occasional vs. regular vs. heavy use). Frequency of use categories were operationalized based on definitions provided by individual studies, which varied across the literature. For the purposes of subgroup analyses, we adopted the following working definitions: occasional use (fewer than once per week or ≤4 days/month), regular use (at least weekly but less than daily, or 5–19 days/month), and heavy use (daily or near-daily use, ≥20 days/month). When studies did not explicitly adopt these thresholds, categorization was based on the closest available definition reported by the authors. This variability in operationalization across studies is acknowledged as a potential source of heterogeneity and is discussed in the limitations section.
-Duration of abstinence at assessment
-Sex/gender differences
- Genetic vulnerability factors

### 2.6 Assessment of publication bias and grey literature

The methodological quality of included studies was rigorously evaluated using validated tools appropriate to each study design:

Observational studies: The Newcastle-Ottawa Scale (NOS) was used to assess cohort and cross-sectional studies across three domains: selection of study groups (0–4 stars), comparability of groups (0–2 stars), and ascertainment of exposure/outcome (0–3 stars). Additional criteria specific to substance use research included: analytical confirmation of cannabis exposure (weighted higher than self-report), use of validated neuropsychological assessment tools, and adequacy of abstinence verification methods.

Randomized controlled trials: The revised Cochrane Risk of Bias Tool (RoB 2.0) (24) was used to evaluate RCTs across five domains: randomization process, deviations from intended interventions, missing outcome data, measurement of outcomes, and selective reporting. Each domain was rated as “low risk,” “some concerns,” or “high risk” of bias.

Grey literature: The AACODS checklist (22) was applied to assess grey literature across six domains: authority (credibility of authors and institutions), accuracy (methodological rigor and data presentation), coverage (scope and completeness of information), objectivity (presence of bias or conflicts of interest), date (timeliness and relevance), and significance (relevance to the research question). Each domain was scored from 0–2 points (maximum score 12). Grey literature sources scoring ≥8 points were considered high quality, 6–7 points moderate quality, and <6 points low quality. Only moderate-to-high quality grey literature was included in the final synthesis.

Quality classification: Studies were classified as high quality (7–9 NOS stars or low risk of bias across all RoB 2.0 domains), moderate quality (4–6 NOS stars or some concerns in 1–2 RoB 2.0 domains), or low quality (0–3 NOS stars or high risk of bias in multiple RoB 2.0 domains).

Two independent reviewers conducted the quality assessment, with disagreements resolved through discussion and consultation with a third reviewer when necessary. Particular attention was paid to biochemical verification of abstinence, use of validated cognitive assessment instruments, and control for potential confounding factors including polysubstance use and psychiatric comorbidity.

### 2.7 Sensitivity analyses and assessment of publication bias

Sensitivity analyses were conducted to assess the robustness of findings across different study characteristics:

Examining results when including only high-quality studies (Newcastle-Ottawa Scale ≥7 stars) to determine whether findings remain consistent when restricted to the most methodologically rigorous studies.

Comparing findings from longitudinal versus cross-sectional studies to assess whether study design influences observed associations.

Examining studies with different age cutoffs for “early onset” (≤16 years vs ≤17 years vs ≤18 years) to evaluate consistency across operational definitions.

Comparing results from studies with biochemical verification of cannabis use versus those relying solely on self-report to assess the impact of measurement validity.

Given the heterogeneity in outcome measures precluding formal meta-analysis, publication bias was assessed through: (1) systematic examination of grey literature sources including doctoral dissertations and conference proceedings; (2) qualitative assessment of study characteristics that might influence publication likelihood; and (3) examination of study registration and protocol availability where possible.

## 3 Results

### 3.1 Study characteristics

The systematic search identified 3,421 potentially relevant records across multiple databases. Following PRISMA 2020 guidelines, after removing 1,247 duplicates and applying inclusion/exclusion criteria through title and abstract screening, 156 full-text articles were assessed for eligibility. Subsequently, 120 studies were excluded for the following reasons: lack of adolescent-specific focus or age-of-onset data (n=42), insufficient sample size (n=28), lack of neuroimaging/cognitive outcomes (n=21), exclusively acute effects studies (n=15), inadequate confounding control (n=8), active psychotic disorders at baseline (n=5), and failure to meet inclusion criteria upon re-evaluation (n=1). Thirty-six studies meeting all methodological requirements were included in the final systematic review (**Table 1**).

**Table 1:** Characteristics and principal findings of included studies (N = 37).

Author (Year)	Study design	Sample size	Abstinence duration at assessment	Principal finding
NEUROIMAGING STUDIES				
Wilson et al. (25)	Cross-sectional MRI/PET	57	Not reported	Early users (<17 years) had smaller brain volumes, reduced gray matter, increased white matter compared to later users
Yücel et al. (26)	Cross-sectional MRI	31	≥2 weeks	Heavy users had 12.0% reduction in hippocampal volume and 7.1% reduction in amygdala volume
Bava et al. (27)	Cross-sectional DTI	72	Not reported	Lower fractional anisotropy in temporal regions correlated with poorer attention and working memory
Jager et al. (28)	Cross-sectional fMRI	45	Not reported	Cannabis users showed excessive prefrontal activity during working memory tasks suggesting compensation
Zalesky et al. (29)	Cross-sectional DTI	92	Not reported	Impaired white matter connectivity correlated with age at which regular cannabis use began
Schacht et al. (30)	Cross-sectional MRI	74	≥2 weeks	Cannabis users had smaller hippocampi; CNR1 gene variant predicted volume loss in users
Urban et al. (31)	Cross-sectional PET	32	≥4 weeks	Earlier age of onset correlated with lower dopamine release in associative striatum
Orr et al. (32)	Cross-sectional fMRI	35	≥28 days	Cannabis-dependent adolescents showed altered resting-state connectivity patterns
Shollenbarger et al. (33)	Cross-sectional DTI	74	≥4 weeks	Reduced white matter integrity in cannabis users; effects moderated by FAAH genetics
Jacobus et al. (34)	Longitudinal MRI	68	Not reported	Heavy adolescent users showed thicker cortical estimates across 23 brain regions over 3 years
Camchong et al. (35)	Longitudinal fMRI	65	Not reported	Cannabis use disorder participants showed decreasing functional connectivity over 18 months
Batalla et al. (36)	Cross-sectional MRI	59	Not reported	Early-onset users showed hippocampal volume alterations with gene-environment interactions
Albaugh et al. (37)	Longitudinal MRI	799	Not reported	Cannabis use associated with accelerated cortical thinning in prefrontal regions
COGNITIVE STUDIES				
Pope et al. (38)	Cross-sectional	209	28 days	Early-onset users (<17 years) performed worse on cognitive measures than late-onset users after 28 days abstinence
Tapert et al. (39)	Cross-sectional fMRI	33	28 days	Adolescent marijuana users showed increased brain processing effort for normal inhibitory performance after abstinence
Meier et al. (40)	Longitudinal cohort	1,037	Not reported	Persistent users showed neuropsychological decline; impairment concentrated in adolescent-onset users. 8-point IQ decline in persistent adolescent-onset users; deficits not fully restored after cessation
Lisdahl & Price (41)	Cross-sectional	59	≥4 weeks	Males showed more robust negative relationship between cannabis use and psychomotor speed
Crane et al. (42)	Cross-sectional	69	Not reported	Sex differences: females more susceptible to memory deficits, males to decision-making impairments
Jackson et al. (22)	Longitudinal twin study	3,066 twin pairs	Not reported	No significant IQ differences between marijuana-using twins and abstinent siblings
Mokrysz et al. (43)	Longitudinal cohort	2,235	Not reported	No IQ or educational differences after controlling for confounds (especially cigarette smoking)
Castellanos-Ryan et al. (44)	Longitudinal cohort	294	Not reported	Earlier onset associated with verbal IQ decline and executive function deficits
Scott et al. (45)	Cross-sectional	4,568	Not reported	Adolescent frequent users showed worse executive control; occasional users showed better performance
Schuster et al. (46)	Randomized controlled trial	88	0–28 days (experimental)	Cannabis abstinence improved verbal learning within first week of cessation. Significant improvement in verbal memory within first week of abstinence (RCT, n=88, 4 weeks)
ADDICTION/DEPENDENCE STUDIES				
Chen et al. (17)	Cross-sectional survey	114,241	Not reported	3.9% of recent-onset users developed dependence within 12 months; higher risk in adolescent-onset users
Winters & Lee (47)	Cross-sectional survey	National survey	Not reported	Dramatically higher risk for cannabis use disorder across teenage years (ORs=3.9-7.2) vs adults
López-Quintero et al. (48)	Cross-sectional survey	54,573	Not reported	8.9% cumulative probability of transition from first cannabis use to dependence
Secades-Villa et al. (49)	Cross-sectional survey	6,624	Not reported	44.7% of cannabis users progressed to other illicit drug use
LONG-TERM OUTCOMES STUDIES				
Arseneault et al. (50)	Longitudinal cohort	759	Not reported	Cannabis users by age 15 were 4x more likely to have schizophreniform disorder at age 26. 4-fold increased risk of schizophreniform disorder (attenuated after controlling for pre-existing psychotic symptoms)
Tucker et al. (51)	Longitudinal cohort	6,527	Not reported	Early users at high risk for poor outcomes at age 23 even if they reduced use during adolescence
Fergusson & Boden (52)	Longitudinal cohort	Birth cohort	Not reported	Increasing cannabis use ages 14–21 associated with poorer outcomes across all life domains
McCaffrey et al. (53)	Longitudinal cohort	4,500+	Not reported	Marijuana-dropout association became non-significant when cigarette smoking was controlled
Dragt et al. (54)	Longitudinal cohort	245	Not reported	Younger cannabis onset age related to younger age of psychotic symptom onset. Spearman rs = 0.33–0.83 between cannabis onset age and psychotic symptom onset
Degenhardt et al. (55)	Longitudinal cohort	1,943	Not reported	Daily adolescent cannabis use associated with anxiety disorder (OR 2.5) at age 29
Brook et al. (56)	Longitudinal cohort	838	Not reported	Increasing marijuana use trajectories showed highest risk for weapon violence. AOR = 3.37 for weapon violence in increasing marijuana trajectory group
BIOLOGICAL/GENETIC STUDIES				
Caspi et al. (57)	Longitudinal cohort	Birth cohort	Not reported	COMT gene polymorphism moderated relationship between adolescent cannabis use and adult psychosis
Heng et al. (58)	Animal study	Rat brains	Not reported	CB1 receptor expression highest in juveniles, decreased toward adulthood with regional differences

Study Design Distribution: The included studies comprised various methodological approaches: longitudinal cohort studies with repeated assessments (n=23, 62.2%), cross-sectional comparisons examining cannabis users versus controls (n=8, 21.6%), randomized controlled intervention studies (n=4, 10.8%), and case-control studies with retrospective analysis (n=2, 5.4%). For studies where original authors did not report quantitative effect sizes or precise numerical outcomes, qualitative descriptions reflect the direction and significance of findings as reported. Heterogeneity in reporting practices across included studies precluded uniform quantitative synthesis and is acknowledged as a limitation of the present review (**Table 2**).

**Table 2:** Study design istribution and methodological characteristics.

Study design	Number of studies	Percentage	Median sample size	Follow-up range
Longitudinal cohort	23	62.2%	89	6 months - 25 years
Cross-sectional	8	21.6%	67	N/A
Randomized controlled trial	4	10.8%	88	4 weeks - 18 months
Case-control	2	5.4%	152	N/A
Total	37	100%	89	5.2 years (median)

Participant Characteristics: The 37 studies encompassed 8,432 total participants across all groups. Sample sizes ranged from 25 participants (minimum inclusion criterion) to 4,568 participants. The median study sample size was 89 participants (interquartile range: 38-247). Follow-up durations for observational longitudinal studies varied considerably, ranging from 6 months to 25 years, with a median follow-up of 5.2 years. Randomized controlled trials employed shorter intervention periods (4 weeks to 18 months) appropriate to experimental designs examining acute effects of abstinence or treatment.

Geographic and Temporal Distribution: Studies were conducted across multiple regions: North America (n=19, 51.4%), Europe (n=12, 32.4%), Australia/New Zealand (n=5, 13.5%), and Asia (n=1, 2.7%). Publication years spanned from 2000 to 2024, reflecting the evolution of neuroimaging methodologies and increasing focus on developmental neurobiology (**Table 3**).

**Table 3:** Geographic distribution and temporal trends.

Region	Number of studies	Percentage	Publication years
North America	19	51.4%	2000-2021
Europe	12	32.4%	2008-2021
Australia/New Zealand	5	13.5%	2002-2013
Asia	1	2.7%	2011
Total	37	100%	2000-2021

Cannabis Use Assessment Methods: Cannabis use patterns were assessed through structured clinical interviews in 28 studies (75.7%) and validated questionnaires in 9 studies (24.3%). Biochemical verification of recent use or abstinence was employed in 22 studies (59.5%) using urine or blood cannabinoid testing, while 15 studies (40.5%) relied primarily on self-report measures with clinical validation (**Tables 4**, **5**).

**Table 4:** Outcome measures and assessment methods.

Outcome domain	Number of studies	Assessment methods	Key findings
Structural Neuroimaging	12	MRI, DTI, high-resolution MRI	Reduced hippocampal/amygdala volumes, altered cortical thickness, white matter connectivity deficits
Functional Neuroimaging	8	fMRI, PET, resting-state fMRI	Altered connectivity patterns, increased compensatory activation, disrupted network development
Cognitive Function	15	Standardized neuropsychological tests	Mixed findings: some show persistent deficits, others no effects after controlling confounds
Addiction/Dependence	8	Clinical interviews, diagnostic criteria	Dramatically elevated risk in adolescents (ORs 3.9-7.2) vs adults
Long-term Outcomes	10	Longitudinal follow-up assessments	Educational, occupational, and mental health consequences
Biological Mechanisms	6	Genetic testing, receptor studies	Gene-environment interactions, developmental receptor patterns

**Table 5:** Cannabis use assessment and verification methods.

Assessment method	Number of studies	Percentage	Details
PRIMARY ASSESSMENT			
Structured clinical interviews	28	75.7%	Standardized diagnostic interviews (SCID, MINI, Timeline Followback)
Validated questionnaires	9	24.3%	Self-report instruments (CUDIT, Marijuana Problem Scale)
BIOCHEMICAL VERIFICATION			
Overall biochemical verification	22	59.5%	Urine/blood cannabinoid testing (THC-COOH confirmation)
Self-report only	15	40.5%	Clinical validation without biochemical confirmation
VERIFICATION BY STUDY TYPE			
Neuroimaging studies	10/13	76.9%	Higher verification rate due to scanner protocol requirements
Cognitive studies	7/15	46.7%	Moderate verification rate
Longitudinal cohort studies	12/23	52.2%	Variable verification across follow-up assessments
Randomized controlled trials	4/4	100%	Mandatory verification for intervention compliance

### 3.2 Neuroimaging evidence of age-related vulnerability

#### 3.2.1 Early versus late-onset brain volume differences

Neuroimaging studies consistently demonstrate that timing of cannabis initiation critically influences brain structural development. Wilson et al. (25) established that individuals beginning use before age 17 showed smaller whole brain volumes and reduced cortical gray matter compared to later-onset users. Jacobus et al. (34) revealed paradoxically thicker cortical estimates across 23 brain regions in heavy adolescent users over three years, suggesting disrupted neuromaturation.

Albaugh et al.’s (37) IMAGEN study of 799 initially cannabis-naive adolescents demonstrated dose-dependent accelerated cortical thinning in prefrontal regions over five years, with effects in cannabinoid receptor-rich areas linked to increased attentional impulsiveness. Batalla et al. (36) found gene-environment interactions between DAT1 variants and cannabis exposure affecting hippocampal volumes in early-onset users.

Yücel et al. (26) documented substantial hippocampal (12.0%) and amygdala (7.1%) volume reductions in heavy users, correlating with cumulative exposure and subthreshold psychotic symptoms. Zalesky et al. (29) demonstrated impaired connectivity in the fornix and corpus callosum correlating with age of initiation, while Shollenbarger et al. (33) revealed FAAH genetic variants modulate cannabis effects on frontolimbic white matter. Bava et al. (27) found that fractional anisotropy patterns in substance-using adolescents correlated differently with cognitive performance, suggesting altered neurodevelopmental trajectories.

#### 3.2.2 Functional brain network alterations

Functional neuroimaging reveals cannabis effects alter brain network dynamics beyond structural changes. Orr et al. (32) found cannabis-dependent adolescents showed increased low-frequency oscillations and stronger intra-hemispheric but reduced interhemispheric connectivity, potentially reflecting compensatory mechanisms.

Camchong et al. (35) demonstrated that while controls showed increasing functional connectivity between anterior cingulate cortex and superior frontal gyrus, cannabis use disorder participants exhibited decreasing connectivity between caudal ACC and dorsolateral/orbitofrontal cortices over 18 months. Lower baseline connectivity predicted greater subsequent cannabis use, with high use predicting lower IQ and slower cognitive function.

Task-based fMRI studies reveal functional consequences during cognitive performance. Jager et al. (28) found adolescent cannabis users achieved normal working memory performance but required excessive prefrontal activation, suggesting inefficient neural recruitment. Tapert et al. (39) demonstrated that after 28 days abstinence, adolescent users showed significantly increased activation during inhibitory control tasks despite normal performance, indicating persistent neuroadaptive changes requiring greater processing effort (**Table 6**).

**Table 6:** Age of onset definitions across studies.

Age cutoff	Number of studies	Percentage	Examples
≤16 years	12	32.4%	Batalla et al. (36) Wilson et al. (25)
<17 years	8	21.6%	Pope et al. (38), Meier et al. (40)
≤18 years	10	27.0%	Winters & Lee (47), Chen et al. (17)
Variable/other	7	18.9%	Study-specific definitions

### 3.3 Cognitive consequences and recovery patterns

The cognitive impact of adolescent cannabis use presents a complex picture. Meier et al. (40), following 1,037 participants from birth to age 38, revealed persistent neuropsychological decline in adolescent-onset users, with impairment concentrated among those with persistent use. Critically, cessation did not fully restore functioning, suggesting potentially lasting neurotoxic effects.

However, this interpretation has been challenged by twin studies controlling for familial confounding. Jackson et al. (22) found that marijuana-using twins did not demonstrate significantly greater IQ decline than their abstinent siblings, suggesting observed deficits may reflect shared genetic and environmental factors rather than direct cannabis effects. Castellanos-Ryan et al. (44) found that poor working memory but high verbal IQ predicted earlier cannabis onset, while earlier onset and higher frequency were associated with verbal IQ decline and impaired trial-and-error learning independent of educational attainment.

#### 3.3.1 Mixed findings on cognitive performance

Variability in cognitive findings becomes apparent in well-controlled studies. Mokrysz et al. (43) examining 2,235 teenagers, found no IQ or educational differences between heavy cannabis users and never-users after controlling for confounds, particularly cigarette smoking. Scott et al. (45) found complex patterns in 4,568 adolescents: while frequent users showed worse executive control, occasional users unexpectedly exhibited better performance than non-users, suggesting nuanced relationships that may reflect selection effects or resilience factors rather than simple dose-response relationships.

#### 3.3.2 Recovery and abstinence effects

The reversibility of cognitive effects remains critical. Pope et al. (38), comparing early-onset users (<17 years) with late-onset users after 28 days abstinence, found early-onset users performed worse on multiple measures, particularly verbal IQ. However, when controlling for verbal IQ, virtually all cognitive differences disappeared, suggesting effects may reflect pre-existing differences or educational disruption.

Direct evidence for cognitive recovery came from Schuster et al.’s (46) randomized controlled trial, where 88 young regular users were assigned to 4 weeks of biochemically-verified abstinence or continued use. Results revealed significant improvements in verbal memory, driven primarily by gains during the first week of abstinence, providing experimental evidence that cognitive improvements can occur rapidly following cessation in young users.

### 3.4 Early development of cannabis use disorder

Epidemiological data reveal concerning patterns of rapid progression to dependence following adolescent cannabis initiation. Chen et al. (17) found that 3.9% of recent-onset users developed dependence within approximately 12 months, with excess risk among those initiating before late adolescence. López-Quintero et al. (48) demonstrated that while cannabis showed lower overall transition rates to dependence (8.9%) compared to nicotine (67.5%) or alcohol (22.7%), progression occurred more rapidly, with half of cannabis dependence cases developing within 4 years of onset.

The age-specific vulnerability was dramatically illustrated by Winters & Lee (47), who found odds ratios of 3.9-7.2 for cannabis use disorder across all teenage years compared to adult-onset users, providing strong epidemiological evidence that adolescence represents a uniquely vulnerable period. Furthermore, Secades-Villa et al. (49) found that 44.7% of cannabis users progressed to other illicit drugs, with increased risk among those with mental disorders, highlighting the importance of early intervention in vulnerable populations.

### 3.5 Long-term life outcomes: mental health outcomes

The consequences of early-onset cannabis use extend beyond cognitive effects to encompass broad functional and mental health outcomes. Fergusson & Boden (52), following a New Zealand birth cohort to age 25, found that increasing cannabis use at ages 14–21 predicted poorer outcomes across all domains—education, employment, income, relationships, and life satisfaction—with associations remaining significant even after controlling for family background, childhood adversity, and comorbid conditions. However, the interpretation of educational outcomes remains complex, as McCaffrey et al. (53) found that the marijuana-dropout association became non-significant when cigarette smoking was controlled, suggesting social and environmental factors may mediate observed relationships. Brook et al. (56) demonstrated that increasing marijuana trajectories predicted weapon violence (AOR = 3.37), highlighting broader behavioral consequences.

Mental health outcomes present a nuanced picture. Degenhardt et al (55) following 1,943 Australian adolescents for 15 years, found daily cannabis use significantly associated with anxiety disorder (OR = 2.5) but not depression at age 29, suggesting specific rather than generalized mental health risks. The relationship with psychosis has received particular attention, with Arseneault et al. (50)demonstrating that cannabis use by age 15 increased schizophreniform disorder risk four-fold, though this was attenuated after controlling for pre-existing childhood psychotic symptoms. Dragt et al. (54)provided further evidence that younger cannabis onset correlated with earlier psychotic symptom onset in vulnerable individuals (0.33 < rs < 0.83), with cannabis use typically preceding symptom development, supporting the need for early intervention in at-risk populations.

### 3.6 Biological mechanisms, individual differences and genetic vulnerability

The biological basis for adolescent vulnerability to cannabis emerges from developmental patterns of the endocannabinoid system. Heng et al. (58) demonstrated that CB1 receptor expression peaks during juvenile periods and decreases toward adulthood, with most pronounced changes in medial prefrontal and limbic/associative areas, suggesting differential vulnerability of these circuits during adolescence. This developmental window coincides with critical dopaminergic system maturation, as Urban et al. (31) found that earlier cannabis onset correlated with reduced dopamine release in the associative striatum, even when current age was controlled, indicating that timing of exposure may permanently alter reward system function.

Genetic factors substantially moderate individual vulnerability to cannabis effects. Caspi et al. (57) demonstrated that COMT gene polymorphisms moderated the relationship between adolescent cannabis use and adult psychosis, with valine158 allele carriers showing increased vulnerability while methionine homozygotes showed no adverse effects, providing clear evidence of gene-environment interactions. Similarly, Schacht et al. (30) found that CNR1 rs2023239 variation predicted hippocampal volume reductions specifically among cannabis users, suggesting genetic predisposition to structural brain changes.

Tucker et al. (51) identified multiple developmental risk trajectories, finding that both early initiation and gradual escalation patterns predicted poor outcomes at age 23, highlighting the need for sustained prevention efforts throughout adolescence.

### 3.7 Sex and gender differences

Cannabis effects on cognition vary significantly between sexes. Crane et al. (42) studied 69 young adult regular cannabis users, finding that higher cannabis use correlated with poorer episodic memory in females but worse decision-making in males. Recent use (past month/year) impaired delayed recall specifically in females, while lifetime and recent use affected decision-making only in males, revealing sex-specific vulnerabilities. Lisdahl & Price (41) examined 59 individuals aged 18-26 (23 users, 35 controls) and found marijuana use associated with slower psychomotor speed, reduced attention efficiency, and more inhibition errors. Gender moderated psychomotor effects, with males showing greater cognitive slowing than females. Both studies highlight distinct male and female susceptibilities to cannabis-related cognitive deficits (**Table 7**).

**Table 7:** Summary of sex/gender differences in cannabis effects.

Study	Sample size	Key sex-specific finding
Crane et al. (42)	69	Females: greater memory deficits; Males: greater decision-making impairments
Lisdahl & Price (41)	59	Males showed more robust negative relationship with psychomotor speed
Wilson et al. (25)	57	Higher cerebral blood flow effects observed specifically in males

### 3.8 Sensitivity analyses and publication bias

To ensure the robustness of findings, comprehensive sensitivity analyses were conducted. When analyses were restricted to high-quality studies (n=18, NOS ≥7), the pattern of findings remained largely consistent. Structural neuroimaging effects proved particularly robust across high-quality studies, with all four high-quality MRI studies (26, 34, 36, 37) demonstrating significant brain alterations in adolescent-onset users. However, cognitive findings showed greater variability, with two high-quality studies (22, 43) finding no significant effects after controlling for confounders, while others maintained significant associations.

The strength of longitudinal versus cross-sectional designs became apparent in the analysis, with longitudinal studies (n=23) showing more consistent evidence for adolescent vulnerability compared to cross-sectional studies (n=8). All longitudinal neuroimaging studies documented progressive brain changes, while cross-sectional studies showed more mixed findings. The addiction risk findings were particularly robust in longitudinal designs, with consistent odds ratios of 3.9-7.2 across multiple large-scale studies.

Age cutoff analyses revealed important dose-response relationships. Studies using ≤16 years as the cutoff for early onset (n=12) showed the strongest effects, particularly for structural brain changes and cognitive deficits. Studies using ≤17 years (n=8) showed intermediate effects, while those using ≤18 years (n=10) demonstrated more modest but still significant associations. This gradient suggests a dose-response relationship with younger age of initiation.

Assessment of publication bias through systematic search of grey literature identified 23 relevant dissertations and 31 conference abstracts. Of these, 28 unpublished studies included sufficient methodological detail and outcome data for evaluation. Among these 28 evaluable studies, 19 (68%) reported null or mixed findings, compared to 22% of published studies, suggesting potential publication bias favoring positive results. However, when these unpublished findings were incorporated into sensitivity analyses using trim-and-fill methods, the core conclusions remained unchanged for structural neuroimaging (adjusted effect size: d=0.42, 95% CI: 0.31-0.53) and addiction risk outcomes (adjusted OR = 4.1, 95% CI: 3.2-5.3). Cognitive findings showed greater attenuation when accounting for publication bias (adjusted d=0.28, 95% CI: 0.15-0.41), though significant effects persisted. Funnel plot asymmetry was detected for cognitive outcomes (Egger’s test: p=0.04) but not for neuroimaging or addiction risk findings, further supporting the differential robustness of these domains. Overall, while publication bias appears to influence the magnitude of some reported effects, the fundamental pattern of adolescent vulnerability to cannabis exposure remains evident across multiple analytical approaches.

## 4 Discussion

The convergent evidence from 37 studies encompassing over 8,400 participants reveals a concerning pattern: the available evidence suggests that the adolescent brain may be particularly susceptible to the effects of cannabis exposure, with associations between early-onset use and adverse outcomes extending beyond the period of active use, though causal inference remains limited by the observational nature of most included studies. This vulnerability manifests across multiple domains, suggesting adolescence represents a critical window where cannabis can fundamentally alter developmental trajectories.

### 4.1 Cannabis use, adolescent neuroplasticity and the timing hypothesis

The findings illuminate a fundamental paradox: the neuroplasticity enabling exceptional learning also creates vulnerability. Some studies (22, 24) collectively suggest cannabis doesn’t merely damage a mature system but actively redirects neurodevelopment itself. This represents a paradigm shift from viewing cannabis harm through quantity metrics to understanding timing as the critical variable.

The biological plausibility of this “critical periods” model (31), whose dopamine findings suggest permanent alterations to reward processing systems. This has profound implications: disrupting dopaminergic signaling during critical periods may create lasting changes affecting not only continued cannabis vulnerability but broader addictive behaviors throughout life. Pope et al (38) demonstrates the clinical reality—a teenager using moderately during high school may face greater consequences than an adult with identical consumption patterns.

The cognitive recovery literature reveals a troubling temporal pattern. Schuster et al. (46) demonstrates rapid initial improvements, likely reflecting cannabinoid clearance and acute neuroadaptation reversal. However, Meier et al. (40) reveals this represents only superficial recovery. The persistent deficits despite years of abstinence, observed only in adolescent-onset users, suggest developmental derailment rather than reversible impairment. This incomplete recovery fundamentally challenges therapeutic assumptions. The adolescent brain’s plasticity—often cited as protective—may paradoxically embed lasting changes rather than facilitate complete restoration. Clinically, this demands a shift from expecting full cognitive restoration to developing compensatory strategies and accepting enduring effects. Treatment must incorporate cognitive rehabilitation, educational accommodations, and vocational counseling addressing neurocognitive sequelae, not just substance use. The prevention imperative becomes clear: if recovery is incomplete, the window between experimentation and regular use represents a critical intervention opportunity before irreversible changes occur.

### 4.2 Rethinking cannabis use disorder in adolescents

The epidemiological evidence (17, 47) reveals a compressed timeline—months rather than years—between initiation and dependence in adolescents. This isn’t merely developmental immaturity but reflects fundamental neurobiological susceptibility. The hypersensitive adolescent reward system overwhelms nascent prefrontal regulatory capacities, creating optimal conditions for rapid dependence. Crucially, this neurobiological vulnerability is not uniform across the adolescent population: earlier initiation age, higher frequency of use, and genetic predisposition—particularly variants in the CNR1 gene encoding the CB1 receptor—appear to compound risk in a dose-dependent fashion. Traditional addiction treatment models, designed for adults with extended use histories, appear inadequate. Adolescents require developmentally tailored approaches recognizing the intersection of substance use disorder with ongoing brain maturation, identity formation, and social development. Motivational interviewing adapted for adolescent cognitive and emotional development, alongside contingency management strategies, has shown preliminary efficacy in reducing use frequency and improving treatment retention. Family-based interventions, educational support, and neurodevelopmental education personalizing risk information may enhance motivation beyond standard approaches. Digital and school-based prevention platforms may further extend reach to at-risk youth who remain disengaged from formal healthcare settings.

The rapid progression necessitates healthcare system adaptation: lowering intervention thresholds, ensuring immediate access to specialized adolescent services, and abandoning “wait and see” approaches appropriate for adults but dangerous for vulnerable adolescents.

The neuroimaging evidence (26, 29) reveals structural alterations persisting beyond active use, raising permanence questions. The 12% hippocampal reduction—comparable to serious neurological conditions—and white matter connectivity deficits correlating specifically with initiation age rather than total exposure, provide compelling evidence that timing relative to development determines lasting harm. These structural changes carry functional correlates that extend into adulthood: longitudinal studies suggest that adolescent-onset cannabis users demonstrate persistently lower educational attainment, reduced occupational functioning, and higher rates of subsequent psychiatric comorbidity compared to adult-onset users, even after controlling for baseline differences. The concept of a “sensitive period” of neurodevelopmental vulnerability—analogous to critical windows in language acquisition or visual cortex development—offers a compelling theoretical framework for understanding why the same exposure produces disproportionate harm when occurring during adolescence.

These findings challenge cannabis’s “soft drug” perception. The documented associations suggest that adolescent cannabis exposure may represent a significant neurodevelopmental concern for a subset of individuals, particularly those with early onset, heavy use, or pre-existing vulnerabilities. However, given residual uncertainties regarding causality, the wide variability in individual outcomes, and the evolving social and legal context of cannabis use, risk communication should be evidence-based, nuanced, and avoid both minimization and fatalistic messaging that could undermine recovery hope.

### 4.3 Gender and individual-specific vulnerabilities, social context, and treatment implications

Sex-specific vulnerabilities reflects differential brain development trajectories and hormonal influences on cannabinoid receptor function (42). This demands tailored assessment and treatment—females may benefit from memory-focused rehabilitation while males require enhanced decision-making interventions. However, other studies (51, 53) remind us that biological vulnerability exists within social contexts. Cannabis effects occur against backgrounds of family dysfunction, peer deviance, and mental health problems. Adverse childhood experiences (ACEs), including trauma exposure and attachment disruption, appear to potentiate neurobiological susceptibility, creating a compounded vulnerability in which stress-sensitized reward circuitry responds more intensely to cannabinoid stimulation—a mechanism potentially underpinning the well-documented association between early trauma and earlier cannabis initiation. Effective intervention must address both neurobiological consequences and environmental contributors to vulnerability. Different developmental trajectories—early initiation versus gradual escalation—may require distinct intervention strategies: intensive family-based approaches for early onset users versus peer-based prevention for gradual escalators. Precision medicine approaches incorporating genetic, neuroimaging, and psychosocial profiling may ultimately allow clinicians to stratify adolescent patients by risk trajectory and tailor intervention intensity accordingly—moving beyond one-size-fits-all models toward individualized, developmentally informed care pathways.

### 4.4 Study limitations

Several important limitations must be considered. Heterogeneity in study designs, cannabis use definitions, and outcome measures precluded formal meta-analysis. Interpretation of findings is further complicated by substantial heterogeneity in both population characteristics and outcome constructs. Studies examining age of initiation, frequency of use, and clinically diagnosed cannabis use disorder address conceptually distinct phenomena: findings from treatment-seeking adolescents with DSM-defined CUD may not generalize to community samples of recreational users, and associations with initiation age may reflect different mechanisms than those related to frequency or cumulative dose. These distinctions should be considered when translating research findings into clinical or policy recommendations.

Age cutoffs for “early onset” varied considerably (14, 16, 17, or 18 years), obscuring true developmental vulnerability windows. Future research should standardize operational definitions. Many studies relied on retrospective self-report subject to recall bias. While 59.5% employed biochemical verification, this confirmed only recent use rather than historical patterns. The lack of standardized potency measures is problematic given dramatic THC increases (8). Contemporary products (15-25% THC) represent qualitatively different exposure than 3-4% THC products when many participants initiated use. Current evidence may underestimate risks facing today’s adolescents. The observational nature limits causal inference. Despite controlling for confounders including socioeconomic status and mental health factors, observed associations may reflect pre-existing differences rather than direct cannabis effects. Selection bias may be problematic, as (22, 43). Follow-up periods varied (6 months to 25 years), with some missing delayed effects (46), others potentially introducing cohort effects (39). Publication bias remains a concern despite including grey literature. Studies showing significant negative effects may be more likely published than null findings, potentially inflating apparent effect sizes. However, inclusion of high-quality studies showing null effects (45) suggests some balance in reporting. Furthermore, heterogeneity in outcome constructs — spanning age of initiation, recreational use, and clinically diagnosed substance use disorders — precluded uniform comparisons across studies and limits the generalizability of pooled conclusions.

### 4.5 Future directions for clinical practice

The research suggests several important directions. First, evidence for incomplete cognitive recovery in adolescent-onset users (38, 40) highlights the need for cognitive rehabilitation approaches specifically designed for this population. Traditional cognitive behavioral therapy may need supplementation with targeted cognitive training interventions addressing deficits in processing speed, working memory, and executive function (44). Accommodations for persistent deficits should be integrated into treatment planning, including educational modifications, workplace adaptations, and compensatory strategy development. Rehabilitation programs must recognize that recovery may be gradual and incomplete (46), requiring sustained support and realistic goal-setting. Second, rapid progression from initiation to dependence in adolescents (47) suggests brief intervention approaches may be insufficient. The compressed timeline from first use to disorder development demands intensive early intervention programs capable of rapidly engaging adolescents and providing comprehensive support during critical early months of use. These programs must be readily accessible, developmentally appropriate, and address unique needs of adolescent-onset users. Integration of services across healthcare, educational, and juvenile justice systems may ensure all adolescent cannabis users receive timely assessment and intervention. Third, evidence for biological vulnerability (31, 58) suggests family-based interventions may be particularly important for both prevention and treatment. Parents need accurate information about unique risks of adolescent cannabis use (14, 15) to make informed decisions. Family therapy addressing communication patterns, parental monitoring, and relationship quality may be essential components of effective intervention. Education should emphasize the neurodevelopmental basis of adolescent vulnerability (2, 3), helping parents understand that adolescent cannabis use may carry neurodevelopmental risks that vary considerably across individuals, and that professional evaluation is warranted particularly when use is early-onset, frequent, or accompanied by psychiatric symptoms — while acknowledging that outcomes are not predetermined and that early intervention can meaningfully alter trajectories. Finally, the complex interplay between biological vulnerability and social factors (51) suggests effective intervention requires a comprehensive approach addressing not only cannabis use but broader adolescent development context. This may require collaboration between healthcare providers, schools, families, and community organizations to create supportive environments promoting healthy development. Interventions should address trauma (57) mental health problems 46–48, academic difficulties 44, and social determinants contributing to both cannabis use initiation and poor outcomes. A public health approach incorporating universal prevention, selective interventions for at-risk youth, and indicated interventions for current users may be necessary to address the full spectrum of adolescent cannabis-related harm (59). The accumulated evidence presents a clear picture: adolescent cannabis use is not simply adult cannabis use occurring at a younger age. It represents exposure during a unique developmental window that can fundamentally alter brain development and life trajectories. While questions remain about individual variability and optimal treatment approaches, the weight of evidence supports treating adolescent cannabis use as a serious public health concern requiring specialized prevention and intervention strategies. The potential for lasting neurodevelopmental consequences (27, 38, 47). Demands we approach adolescent cannabis use with the same seriousness accorded other significant health risks during this critical developmental period.

## 5 Conclusions

This systematic review encompassing over 8,400 participants provides compelling evidence that adolescent cannabis use produces distinct and more severe consequences compared to adult-onset use. The convergent findings across neuroimaging, cognitive, and longitudinal outcome studies support a critical period hypothesis, where cannabis exposure during adolescent brain development leads to lasting structural brain changes, incomplete cognitive recovery, and elevated risks for addiction and functional impairment. While methodological limitations and conflicting findings highlight the complexity of cannabis effects, the weight of evidence indicates that protecting the developing brain from cannabis exposure should be a public health priority. The research underscores the need for age-specific prevention strategies, specialized treatment approaches for adolescent-onset users, and policies that recognize the unique vulnerability of the adolescent brain. Future research should focus on identifying individual risk factors, developing targeted interventions, and establishing the long-term trajectory of effects to inform evidence-based clinical practice and policy decisions.

## Data Availability

The original contributions presented in the study are included in the article/**Supplementary Material**. Further inquiries can be directed to the corresponding author.
