# Supplementary material for: How adolescent cannabis use reshapes the developing brain — a systematic review
Source: Front Psychiatry. 2026 Apr 27;17:1822300. doi: 10.3389/fpsyt.2026.1822300 (PMC13158221; doi:10.3389/fpsyt.2026.1822300)
Supplement: Supplementary file 1 [file Table1.docx]

**Appendix A: PRISMA 2020 Checklist**

| Section and Topic | Item # | Checklist item | Location where item is reported |
| --- | --- | --- | --- |
| TITLE |  |  |  |
| Title | 1 | Identify the report as a systematic review. | Page 1 |
| ABSTRACT |  |  |  |
| Abstract | 2 | See the PRISMA 2020 for Abstracts checklist. | Page 1 |
| INTRODUCTION |  |  |  |
| Rationale | 3 | Describe the rationale for the review in the context of existing knowledge. | Pages 3-5 |
| Objectives | 4 | Provide an explicit statement of the objective(s) or question(s) the review addresses. | Page 5 |
| METHODS |  |  |  |
| Eligibility criteria | 5 | Specify the inclusion and exclusion criteria for the review and how studies were grouped for the syntheses. | Pages 6-7 |
| Information sources | 6 | Specify all databases, registers, websites, organisations, reference lists and other sources searched or consulted to identify studies. Specify the date when each source was last searched or consulted. | Page 6 |
| Search strategy | 7 | Present the full search strategies for all databases, registers and websites, including any filters and limits used. | Page 6 |
| Selection process | 8 | Specify the methods used to decide whether a study met the inclusion criteria of the review, including how many reviewers screened each record and each report retrieved, whether they worked independently, and if applicable, details of automation tools used in the process. | Page 7 |
| Data collection process | 9 | Specify the methods used to collect data from reports, including how many reviewers collected data from each report, whether they worked independently, any processes for obtaining or confirming data from study investigators, and if applicable, details of automation tools used in the process. | Pages 7-8 |
| Data items | 10a | List and define all outcomes for which data were sought. Specify whether all results that were compatible with each outcome domain in each study were sought (e.g. for all measures, time points, analyses), and if not, the methods used to decide which results to collect. | Page 8 |
| Data items | 10b | List and define all other variables for which data were sought (e.g. participant and intervention characteristics, funding sources). Describe any assumptions made about any missing or unclear information. | Page 8 and Appendix B |
| Study risk of bias assessment | 11 | Specify the methods used to assess risk of bias in the included studies, including details of the tool(s) used, how many reviewers applied the tool and whether they worked independently, and if applicable, details of automation tools used in the process. | Pages 8-9 |
| Effect measures | 12 | Specify for each outcome the effect measure(s) (e.g. risk ratio, mean difference) used in the synthesis or presentation of results. | Page 9 |
| Synthesis methods | 13a | Describe the processes used to decide which studies were eligible for each synthesis (e.g. tabulating the study intervention characteristics and comparing against the planned groups for each synthesis (original plan)). | Page 9 |
| Synthesis methods | 13b | Describe any methods required to prepare the data for presentation or synthesis, such as handling of missing summary statistics, or data conversions. | Page 9 |
| Synthesis methods | 13c | Describe any methods used to tabulate or visually display results of individual studies and syntheses. | Pages 9, Tables 1-7 |
| Synthesis methods | 13d | Describe any methods used to synthesize results and provide a rationale for the choice(s). If meta-analysis was performed, describe the model(s), method(s) to identify the presence and extent of statistical heterogeneity, and software package(s) used. | Page 9 |
| Synthesis methods | 13e | Describe any methods used to explore possible causes of heterogeneity among study results (e.g. subgroup analysis, meta-regression). | Pages 9-10 |
| Synthesis methods | 13f | Describe any sensitivity analyses conducted to assess robustness of the synthesized results. | Pages 9-10 |
| Reporting bias assessment | 14 | Describe any methods used to assess risk of bias due to missing results in a synthesis (arising from reporting biases). | Page 10 |
| Certainty assessment | 15 | Describe any methods used to assess certainty (or confidence) in the body of evidence for an outcome. | Pages 8-9 |
| RESULTS |  |  |  |
| Study selection | 16a | Describe the results of the search and selection process, from the number of records identified in the search to the number of studies included in the review, ideally using a flow diagram. | Page 11 |
| Study selection | 16b | Cite studies that might appear to meet the inclusion criteria, but which were excluded, and explain why they were excluded. | Page 11 |
| Study characteristics | 17 | Cite each included study and present its characteristics. | Pages 11-12, Table 1 |
| Risk of bias in studies | 18 | Present assessments of risk of bias for each included study. | Pages 12, Table 6 |
| Results of individual studies | 19 | For all outcomes, present, for each study: (a) summary statistics for each group (where appropriate) and (b) an effect estimate and its precision (e.g. confidence/credible interval), ideally using structured tables or plots. | Pages 13-32, Tables 1-7 |
| Results of syntheses | 20a | For each synthesis, briefly summarise the characteristics and risk of bias among contributing studies. | Pages 12-32 |
| Results of syntheses | 20b | Present results of all statistical syntheses conducted. If meta-analysis was done, present for each the summary estimate and its precision (e.g. confidence/credible interval) and measures of statistical heterogeneity. If comparing groups, describe the direction of the effect. | Pages 32-33 (Sensitivity analyses) |
| Results of syntheses | 20c | Present results of all investigations of possible causes of heterogeneity among study results. | Pages 32-33 |
| Results of syntheses | 20d | Present results of all sensitivity analyses conducted to assess the robustness of the synthesized results. | Pages 32-33 |
| Reporting biases | 21 | Present assessments of risk of bias due to missing results (arising from reporting biases) for each synthesis assessed. | Page 33 |
| Certainty of evidence | 22 | Present assessments of certainty (or confidence) in the body of evidence for each outcome assessed. | Pages 34-39 (Discussion) |
| DISCUSSION |  |  |  |
| Discussion | 23a | Provide a general interpretation of the results in the context of other evidence. | Pages 34-39 |
| Discussion | 23b | Discuss any limitations of the evidence included in the review. | Pages 39-40 |
| Discussion | 23c | Discuss any limitations of the review processes used. | Pages 39-40 |
| Discussion | 23d | Discuss implications of the results for practice, policy, and future research. | Pages 40-41 |
| OTHER INFORMATION |  |  |  |
| Registration and protocol | 24a | Provide registration information for the review, including register name and registration number, or state that the review was not registered. | Page 6 |
| Registration and protocol | 24b | Indicate where the review protocol can be accessed, or state that a protocol was not prepared. | Page 6 |
| Registration and protocol | 24c | Describe and explain any amendments to information provided at registration or in the protocol. | Not applicable |
| Support | 25 | Describe sources of financial or other support for the review, and the role of the funders or sponsors in the review. | Page 41 |
| Competing interests | 26 | Declare any competing interests of review authors. | Page 41 |
| Availability of data, code and other materials | 27 | Report which of the following are publicly available and where they can be found: template data collection forms; data extracted from included studies; data used for all analyses; analytic code; any other materials used in the review. | Page 41 |

**Appendix B: Data Extraction Variables**

| Category | All Studies | Neuroimaging Studies | Cognitive Studies | Longitudinal Studies |
| --- | --- | --- | --- | --- |
| STUDY CHARACTERISTICS |  |  |  |  |
| Study identification | First author, publication year, journal | ✓ | ✓ | ✓ |
| Study design | Cross-sectional, longitudinal cohort, RCT, case-control | ✓ | ✓ | ✓ |
| Country/Region | Geographic location of study | ✓ | ✓ | ✓ |
| Setting | Clinical, community, university-based | ✓ | ✓ | ✓ |
| Follow-up duration | Duration of follow-up period | - | - | ✓ |
| Loss to follow-up | Percentage and reasons for dropout | - | - | ✓ |
| PARTICIPANT CHARACTERISTICS |  |  |  |  |
| Sample size | Total N, N per group | ✓ | ✓ | ✓ |
| Age | Mean age, age range, age at assessment | ✓ | ✓ | ✓ |
| Sex/Gender | Male/female distribution, percentage | ✓ | ✓ | ✓ |
| Ethnicity/Race | Demographic composition when reported | ✓ | ✓ | ✓ |
| Socioeconomic status | Education level, income, parental SES | ✓ | ✓ | ✓ |
| IQ/Cognitive baseline | Pre-exposure or baseline cognitive measures | ✓ | ✓ | ✓ |
| CANNABIS USE VARIABLES |  |  |  |  |
| Age of initiation | Age at first cannabis use | ✓ | ✓ | ✓ |
| Early onset definition | Definition used (≤16, <17, ≤18 years) | ✓ | ✓ | ✓ |
| Frequency of use | Daily, weekly, occasional use patterns | ✓ | ✓ | ✓ |
| Duration of use | Total years of cannabis use | ✓ | ✓ | ✓ |
| Cumulative exposure | Total lifetime joints/episodes | ✓ | ✓ | ✓ |
| Cannabis potency | THC content when available | ✓ | ✓ | ✓ |
| Route of administration | Smoking, vaporizing, edibles | ✓ | ✓ | ✓ |
| Abstinence duration | Time since last use at assessment | ✓ | ✓ | ✓ |
| Cannabis use disorder | DSM/ICD criteria for dependence | ✓ | ✓ | ✓ |
| CONTROL GROUP |  |  |  |  |
| Control type | Healthy controls, non-using peers | ✓ | ✓ | ✓ |
| Matching criteria | Age, sex, education, SES matching | ✓ | ✓ | ✓ |
| Cannabis exposure | Never-users vs. minimal exposure | ✓ | ✓ | ✓ |
| CONFOUNDING VARIABLES |  |  |  |  |
| Alcohol use | Frequency, quantity, age of onset | ✓ | ✓ | ✓ |
| Tobacco use | Cigarette smoking patterns | ✓ | ✓ | ✓ |
| Other substance use | Illicit drugs, prescription medications | ✓ | ✓ | ✓ |
| Mental health history | Psychiatric diagnoses, treatment history | ✓ | ✓ | ✓ |
| Family history | Substance use, mental health in relatives | ✓ | ✓ | ✓ |
| NEUROIMAGING MEASURES |  |  |  |  |
| Imaging modality | MRI, fMRI, DTI, PET specifications | - | ✓ | - |
| Scanner details | Tesla strength, manufacturer | - | ✓ | - |
| Acquisition parameters | TR, TE, voxel size, sequences used | - | ✓ | - |
| Processing software | SPM, FSL, FreeSurfer versions | - | ✓ | - |
| Structural measures | Regional volumes, cortical thickness | - | ✓ | - |
| White matter measures | FA, MD, radial/axial diffusivity | - | ✓ | - |
| Functional measures | BOLD activation, connectivity metrics | - | ✓ | - |
| Regions of interest | Predefined ROIs, whole-brain analyses | - | ✓ | - |
| COGNITIVE MEASURES |  |  |  |  |
| Assessment battery | Standardized neuropsychological tests | - | ✓ | ✓ |
| Intelligence measures | WAIS, WISC, estimated IQ | - | ✓ | ✓ |
| Memory domains | Verbal, visual, working memory tasks | - | ✓ | ✓ |
| Executive function | Attention, inhibition, cognitive flexibility | - | ✓ | ✓ |
| Processing speed | Psychomotor, reaction time measures | - | ✓ | ✓ |
| Language function | Verbal fluency, comprehension tasks | - | ✓ | ✓ |
| Visuospatial abilities | Spatial processing, construction tasks | - | ✓ | ✓ |
| ADDICTION/DEPENDENCE OUTCOMES |  |  |  |  |
| Diagnostic criteria | DSM-IV, DSM-5, ICD criteria used | ✓ | - | ✓ |
| Assessment method | Clinical interview, self-report scales | ✓ | - | ✓ |
| Time to dependence | Months/years from initiation to CUD | ✓ | - | ✓ |
| Severity measures | Mild, moderate, severe classifications | ✓ | - | ✓ |
| Withdrawal symptoms | Physical, psychological withdrawal | ✓ | - | ✓ |
| Treatment seeking | Help-seeking behaviors, treatment entry | ✓ | - | ✓ |
| LONG-TERM OUTCOMES |  |  |  |  |
| Educational outcomes | High school completion, academic performance | ✓ | - | ✓ |
| Occupational function | Employment status, job performance | ✓ | - | ✓ |
| Mental health outcomes | Depression, anxiety, psychosis incidence | ✓ | - | ✓ |
| Social functioning | Relationship quality, social support | ✓ | - | ✓ |
| Legal problems | Arrests, legal consequences | ✓ | - | ✓ |
| Other substance progression | Transition to other illicit drugs | ✓ | - | ✓ |
| BIOLOGICAL MEASURES |  |  |  |  |
| Genetic factors | COMT, CNR1, DAT1, FAAH polymorphisms | ✓ | ✓ | ✓ |
| Biochemical verification | Urine, blood, saliva cannabinoid testing | ✓ | ✓ | ✓ |
| Hormonal measures | Testosterone, cortisol levels | ✓ | ✓ | ✓ |
| Neurotransmitter function | Dopamine, GABA system measures | ✓ | ✓ | ✓ |
| STATISTICAL METHODS |  |  |  |  |
| Primary analysis | Statistical tests used for main outcomes | ✓ | ✓ | ✓ |
| Effect sizes | Cohen's d, eta-squared, odds ratios | ✓ | ✓ | ✓ |
| Confidence intervals | 95% CIs for effect estimates | ✓ | ✓ | ✓ |
| P-values | Significance levels, multiple comparisons | ✓ | ✓ | ✓ |
| Sample size calculation | Power analysis, justification of N | ✓ | ✓ | ✓ |
| Missing data handling | Imputation methods, sensitivity analyses | ✓ | ✓ | ✓ |
| Covariate adjustment | Variables controlled in analyses | ✓ | ✓ | ✓ |
| QUALITY INDICATORS |  |  |  |  |
| Biochemical verification | Cannabis use confirmed by testing | ✓ | ✓ | ✓ |
| Blinding procedures | Assessor blinding to group status | ✓ | ✓ | ✓ |
| Standardized assessments | Validated instruments used | ✓ | ✓ | ✓ |
| Inter-rater reliability | Kappa coefficients, ICC values | ✓ | ✓ | ✓ |
| Selection bias control | Recruitment methods, representativeness | ✓ | ✓ | ✓ |
| Confounding control | Adequate adjustment for key confounders | ✓ | ✓ | ✓ |

**Appendix C. Quality Assessment Results**

| Quality Rating | Number of Studies | Percentage | Newcastle-Ottawa Scale Score Range |
| --- | --- | --- | --- |
| High quality | 18 | 48.6% | 7-9 stars |
| Moderate quality | 15 | 40.5% | 4-6 stars |
| Low quality | 4 | 10.8% | 0-3 stars |
